# Identification and Validation of an Immune-Related eRNA Prognostic Signature for Hepatocellular Carcinoma

**DOI:** 10.3389/fgene.2021.657051

**Published:** 2021-06-11

**Authors:** Shenglan Cai, Xingwang Hu, Ruochan Chen, Yiya Zhang

**Affiliations:** ^1^Hunan Key Laboratory of Viral Hepatitis, Xiangya Hospital, Central South University, Changsha, China; ^2^Department of Infectious Disease, Xiangya Hospital, Central South University, Changsha, China; ^3^Department of Dermatology, Xiangya Hospital, Central South University, Changsha, China; ^4^National Clinical Research Center for Geriatric Disorders, Xiangya Hospital, Central South University, Changsha, China

**Keywords:** non-coding RNA, immune signature, overall survival, tumor microenvironment, hepatocellular carcinoma

## Abstract

**Background:**

Enhancer RNAs (eRNAs) are intergenic long non-coding RNAs (lncRNAs) that participate in the progression of malignancies by targeting tumor-related genes and immune checkpoints. However, the potential role of eRNAs in hepatocellular carcinoma (HCC) is unclear. In this study, we aimed to construct an immune-related eRNA prognostic model that could be used to prospectively assess the prognosis of patients with HCC.

**Methods:**

Gene expression profiles of patients with HCC were downloaded from The Cancer Genome Atlas (TCGA). The eRNAs co-expressed from immune genes were identified as immune-related eRNAs. Cox regression analyses were applied in a training cohort to construct an immune-related eRNA signature (IReRS), that was subsequently used to analyze a testing cohort and combination of the two cohorts. Kaplan-Meier and receiver operating characteristic (ROC) curves were used to validate the predictive effect in the three cohorts. Gene Set Enrishment Analysis (GSEA) computation was used to identify an IReRS-related signaling pathway. A web-based cell type identification by estimating relative subsets of RNA transcripts (CIBERSORT) computation was used to evaluate the relationship between the IReRS and infiltrating immune cells.

**Results:**

A total of sixty-four immune-related eRNAs (IReRNAs) was identified in HCC, and 14 IReRNAs were associated with overall survival (OS). Five IReRNAs were used for constructing an immune-related eRNA signature (IReRS), which was shown to correlate with poor survival and to be an independent prognostic biomarker for HCC. The GSEA results showed that the IReRS was correlated to cancer-related and immune-related pathways. Moreover, we found that IReRS was correlated to infiltrating immune cells, including CD8^+^ T cells and M0 macrophages. Finally, differential expressions of the five risk IReRNAs in tumor tissues vs. adjacent normal tissues and their prognostic values were verified, in which the *AL445524.1* may function as an oncogene that affects prognosis partly by regulating CD4-CLTA4 related genes.

**Conclusion:**

Our results suggest that the IReRS could serve as a biomarker for predicting prognosis in patients with HCC. Additionally, it may be correlated to the tumor immune microenvironment and could also be used as a biomarker in immunotherapy for HCC.

## Introduction

Hepatocellular carcinoma (HCC) is the main type of primary liver cancer, which is the fifth most common cancer and the second most frequent cause of cancer-associated deaths (European Association for the Study of the Liver, 2018). A poor prognosis in patients with HCC was in part a result of incomplete understanding of epigenetic and heterogeneous gene alterations to reliably obtain predictable biomarkers (El-Serag and Rudolph, 2007). A liable assessment model is needed to improve survival prognosis and appropriate treatment decision in patients with HCC.

HCC is closely related to chronic liver inflammation and is considered to be a typical immunogenic cancer (Pardee and Butterfield, 2012). Tumor associated macrophages (TAM) and myeloid-derived suppressive cells (MDSC) in the HCC microenvironment release cytokines, such as IL-6 and tumor necrosis factor (TNF), thereby inducing cancer cell proliferation and inhibiting apoptosis through activation of NF-κB and Stat3 (Iliopoulos et al., 2009; Yu et al., 2009). Immunosuppressive cells in the HCC tumor microenvironment can interfere with immune monitoring, leading to tumor’s immune evasion or immune escape (Zamarron and Chen, 2011). A scoring of a gene ontology and pathway analysis has shown that HCC-related genes are also closely related to genes involving in immune microenvironment (Ge et al., 2020). Immunotherapies targeting co-inhibitory receptors (i.e., CTLA4 and PD1) have been used to treat some cancers, such as pulmonary cancers and metastatic melanoma (Wolchok, 2015; Chikuma, 2017). Therefore, an immune-related prognostic model for HCC is worth exploring.

Long non-coding RNAs (lncRNAs) act as important regulators in the tumor progression of various cancers, including HCC (Shi et al., 2016; Li et al., 2017; Yu et al., 2020). Enhancer RNA (eRNA) is a type of lncRNA transcribed from intergenic enhancers, and exists widely in most human cells and tissues (Chen et al., 2017). Emerging evidence suggests that eRNAs are integral components of enhancer function. Recent studies have revealed the involvement of eRNAs in the progression of cancers (Liu et al., 2018; Zhang Z. et al., 2019). For example, eRNA *KLK3e* is able to activate KLK3 and to alter androgen receptor dependent gene expression, and subsequently to promote cancer cell proliferation in a prostate cancer line (Hsieh et al., 2014); *NET1e* is highly expressed in breast invasive carcinoma, and is associated with poor prognosis (Li et al., 2015); *TAOK1e* is related to the overall survival of patients with clear cell renal cell carcinoma; *EN1e* is highly expressed in breast carcinoma; *CELF2e* is highly expressed in stage III stomach adenocarcinoma; and *APH1A* is highly expressed in grade 3 HCC (Zhang Z. et al., 2019). Studies have shown that lncRNAs are also closely related to the immune microenvironment of HCC (Hong et al., 2020; Kong et al., 2020). However, the prognostic role of immune-related eRNAs in HCC remains unclear.

In this study, we explored the prognostic significance of immune-related eRNAs (IReRNAs) in HCC from The Cancer Genome Atlas (TCGA) database. We screened IReRNAs to construct a novel eRNA prognostic model, termed immune-related eRNA signature (IReRS), which was shown here to correlate with poor survival and to be an independent prognostic factor. Moreover, the IReRS was correlated to immune-cell infiltration. Finally, a risk eRNA, *AL445524.1*, was identified as a key HCC-related eRNA and closely related to tumor immunity by regulating downstream genes.

## Materials and Methods

### Identification of Immune-Related eRNAs

A total of 567 immune-related genes (IRGs) in HCC from the Molecular Signature Database v4. 0 were identified by GSEA. The expression data and clinical data of HCC were downloaded from TCGA. This dataset contains 371 HCC tumor samples and 54 adjacent normal samples. Samples without complete survival information were removed. Ultimately, 366 HCC samples were included for subsequent analysis. eRNAs were predicted using PreSTIGE as previously described (Murakami et al., 2015; Gu et al., 2019). Pearson correlation analysis was carried out between the expression level of eRNAs and the expression level of IRGs to identify immune-related eRNAs (Pearson correlation coefficient > 0.4, *p* < 0. 001).

### Random Grouping of Data

The expression level of immune-related eRNAs (HtSeq-FPKM) and clinical data of 366 HCC samples were downloaded from TCGA on June 16, 2020. A total of 1,580 eRNAs were identified in HCC tissues from TCGA dataset. Using Pearson correlation analysis, 64 Immune-related eRNAs were identified in HCC (*R* > 0.4, *p* < 0.001). The 366 samples were randomly divided into two groups, a training cohort (60%) and a testing cohort (40%), using the R package “caret.” The clinical characteristics of the patients with HCC are shown in Table 1.

**TABLE 1:** Clinical features of the hepatocellular carcinoma patients in each cohort.

**Variables**	**Training cohort (*n* = 224)**	**Entire TCGA cohort (*n* = 366)**	***p***
**Age**			
≤ 55	69	122	0.524
>55	155	244	
**Race**			
Asia	90	155	0.819
Black or African American	10	17	
White	115	183	
Unknown	9	10	
American Indian/Alaska Native	0	1	
**Gender**			
Female	69	119	0.665
Male	115	247	
**Tumor stage**			
Stage I	103	170	0.994
Stage II	53	85	
Stage III	52	83	
Stage IV	3	4	
Unkown	13	24	
**Grade**			
G1	28	55	0.658
G2	119	176	
G3	65	118	
G4	7	12	
Unkown	5	5	

### Construction of a Prognostic IReRS

**FIGURE 1:**
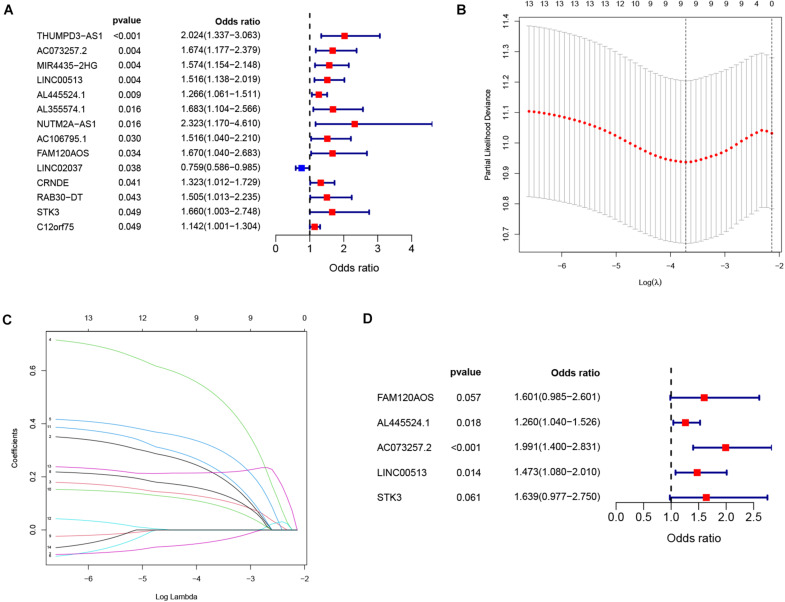
Identification of immune-related eRNAs from hepatocellular carcinoma. **(A)** Univariate Cox regression models identified 14 immune-related eRNAs that are associated with OS. Two analyses **(B,C)** by a lasso regression further fine-tuned the selection of immune-related eRNAs. **(D)** Multivariate Cox regression analysis identified five immune-related eRNAs for the construction of a prognostic model.

We performed Cox analysis and lasso regression to establish the IReRS in the training cohort, and then verified the IReRS in both the testing cohort and the entire cohort. The risk score was calculated using eRNA expression and coefficient values (Gu et al., 2019). Fourteen eRNAs were identified as being associated with overall survival (OS) by the univariate Cox regression models (Figure 1A). In addition, the least absolute shrinkage and selection operator (LASSO) regression analysis was performed (Figures 1B,C), and finally, 5 immune-related risk eRNAs (*FAM120AOS, AL445524.1*, *AC073257.2*, *LINC00513, STK3*) were identified to construct the risk model by the multivariate Cox regression analysis (Figure 1D). The formula to calculate the risk score for the IReRS is: 0.4705 × expression of *FAM120AOS* + 0.2308 × expression of *AL445524.1* + 0.6886 × expression of *AC073257.2* + 0.3875 × expression of *LINC00513* + *0.4941* × expression of *STK3.*

The median risk score in the training cohort served as the cutoff value, which was applied to divide the entire cohort into a low-risk group and a high-risk group. For survival analysis, Kaplan–Meier survival curves were constructed for both the low and high-risk groups in all three cohorts using R package “survival,” and two-sided log-rank test of *p* < 0.05 was considered statistically significantly.

The prognostic value of the IReRS was checked using the R package “survival.” Furthermore, a survival receiver operating characteristic (ROC) curve was constructed using the R package “survival ROC” to verify the prognostic performance.

A nomogram was constructed from the risk score and other clinical parameters in each cohort. ROC curves were used to compare the prognostic value of risk scores with other clinical features using R software “ROC package.”

### Gene Set Enrichment Analysis

We analyzed the enrichment terms in the entire TCGA cohort using GSEA software version 4.1.1 (Cambridge, MA, United States) to reveal pathways related to IReRS (Mootha et al., 2003; Subramanian et al., 2005). The gene sets of “ h. all. v7.1. symbols. gmt (cancer hallmarks) and c7. all. v7.1. symbols. gmt (Immunologic signatures) ” were selected for GSEA analysis, and a *p* < 0.05 along with a false discovery rate < 0.05 was considered statistically significant.

### Infiltrating Immune Cells in HCC

The data on infiltration of immune cells in HCC were obtained using CIBERSORT (Newman et al., 2015). Differences of infiltrating immune cells in high- and low-risk HCC samples were examined by Wilcoxon. Using the R ‘‘ESTIMATE’’ software package, we calculated tumor microenvironment score. DNA methylation Stemness score (DNASS) and RNA Stemness score (RNASS) were downloaded from the UCSC database^1^. The correlation between risk score and tumor microenvironment and tumor stem cell score in HCC was calculated using the R software package (“CorrPlot”).

### Immune Checkpoint Blockade (ICB) Analysis in HCC

Six genes were previously reported as key targets of immune checkpoint inhibitors: TIM-3, IDO1, CTLA-4, PD-1, PD-L1, and PD-L2 (Goodman et al., 2017; Kim et al., 2017; Nishino et al., 2017). The difference of immune checkpoint inhibitor treatment in malignant tumor is related to the difference of immune checkpoint gene expression (Gu et al., 2019). The correlation between the six immune checkpoint inhibitors and our immune-related signature was analyzed to explore the possible role of the IReRNA and IReRS in HCC ICB therapy by Spearman’s correlation coefficient.

### The Expression of the Immune-Related Risk eRNAs

The expression level of risk eRNAs were identified from TCGA datasets. The R package ‘‘survival’’ was then used to study the prognostic values of risk eRNAs in HCC. The tSNE analysis of risk eRNA-associated genes was carried out using web tools^2^.

### Statistical Analysis

The Wilcoxon signed-rank test was used for the correlation analysis between IReRS and the clinical characteristics of patients with HCC. The relationships among IReRS and immune cells, immune cell markers, and ICB were analyzed by Spearman’s correlation coefficient. Kaplan-Meier curves were used for survival analysis. Among 567 IRGs, the genes associated with *AL445524.1* were screened by Pearson correlation analysis.

## Results

### Validation of the IReRS in TCGA

**FIGURE 2:**
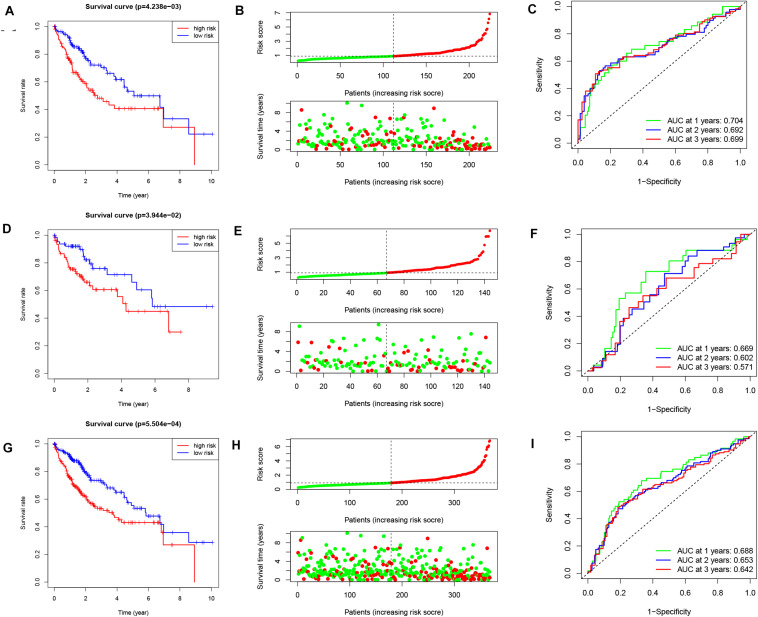
Kaplan–Meier survival analysis, risk score analysis and ROC curve analysis. Kaplan-Meier survival curve, risk score, and ROC curve analysis of the immune-related eRNA signature were illustrated within the training cohort [**(A–C)**, respectively], within the testing cohort [**(D–F)**, respectively], and within the entire cohort [**(G–I)**, respectively].

Patients in high-risk group had a significantly worse OS in the training cohort, testing cohort and entire cohort (Figures 2A,D,G, *p* = 4.238e-3, *p* = 3.944e-2, and *p* = 5.504e-4, respectively). The survival time decreased as the risk score increased (Figures 2B,E,H). The computed area under the curve (AUC) values of the IReRS were 0.704, 0.692, and 0.699 for survival time of 1, 2, and 3 years, respectively, in the training cohort (Figure 2C), and the corresponding values of 0.669, 0.602, and 0.571 in the testing cohort (Figure 2F) and of 0.688, 0.653, and 0.642 in the entire cohort (Figure 2I) were also comparable. The results indicate that the model has good predictive ability, specificity, and sensitivity.

### Correlations Between the Clinicopathologic Characteristics and the IReRS in HCC

**FIGURE 3:**
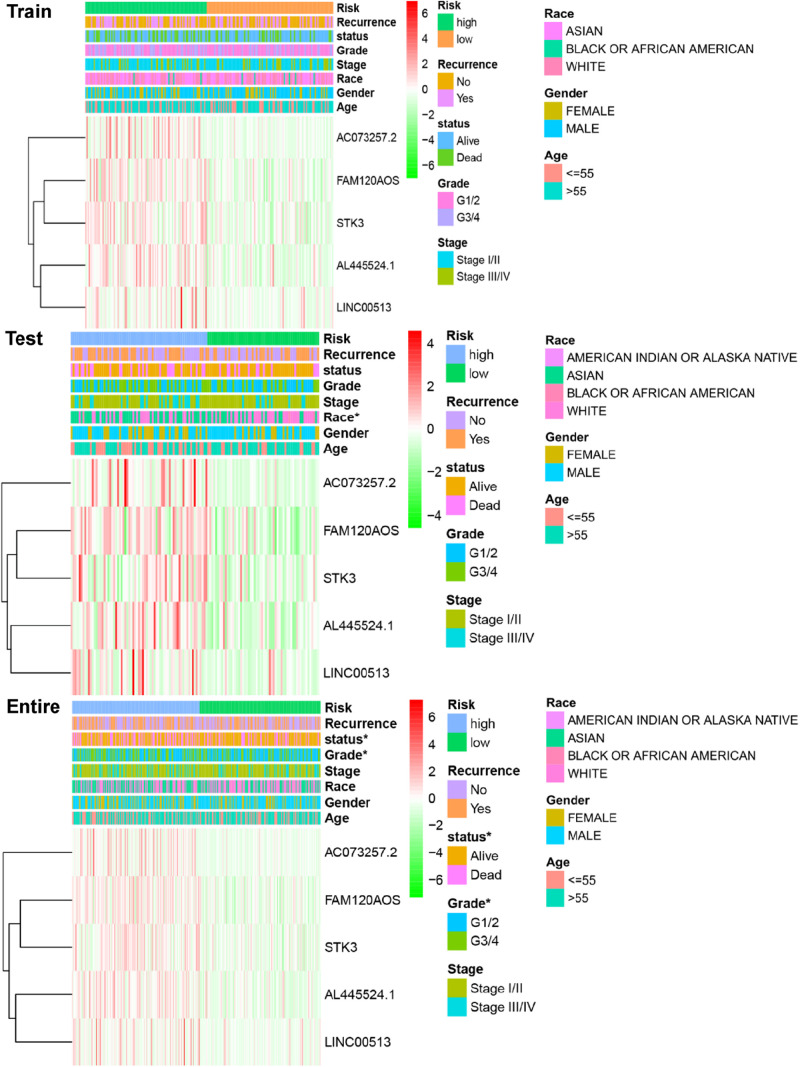
Relationships between the risk score and clinical characteristics of HCC in three cohorts. Significant associations of risk scores with patients’ survival status and histological grade in the entire cohort (bottom panel) were observed. *p < 0.05.

We next analyzed the relationships between IReRS and clinical characteristics, including age (≤55, >55 years old), race, gender, survival status, pathological tumor stage, recurrence, and histological grade by Pearson correlation analysis. The IReRS risk score, however, did not significantly correlate with clinical characteristics in training or in testing cohort. In the entire cohort, the risk score was found to be significantly correlated with histological grade and survival status (Figure 3).

### Identification of Independent Prognostic Factors

**FIGURE 4:**
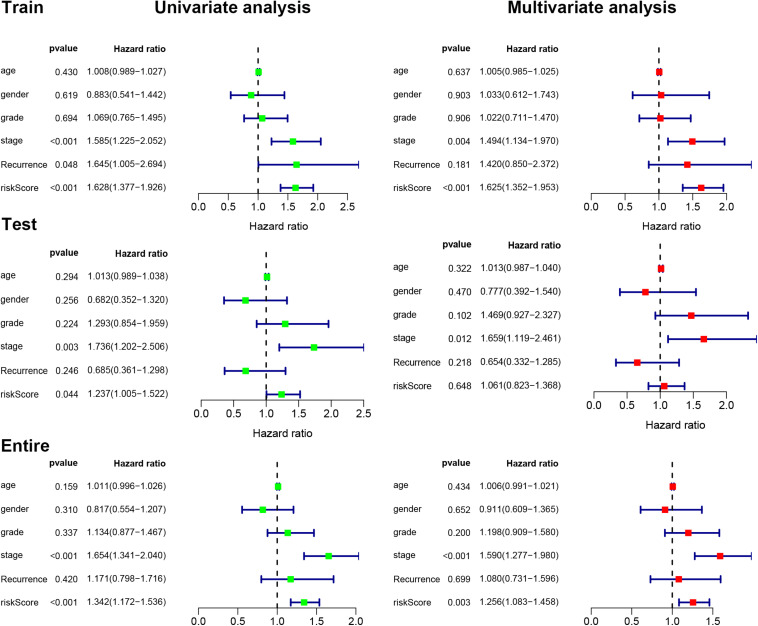
Univariate and multivariate Cox regression analyses. Univariate and multivariate Cox regression analyses illustrated the clinical features within the training, testing, and entire cohorts.

We next used univariate and multivariate Cox regression analyses to examine the independent prognostic role of IReRS with clinical characteristics, including age, gender, histological grade, pathological tumor stage, recurrence, and riskscore. As shown in Figure 4, univariate and multivariate Cox regression analyses showed that the pathological tumor stage and risk score were independent prognostic factors for OS. Multivariate Cox regression analysis further identified the IReRS to be an independent prognostic factor for OS in the training cohort and the entire cohort with an HR of 1. 625 (1.352–1.953) and 1.256 (1.083–1.458), respectively. The results suggest that IReRS, in addition to the tumor stage and risk score, could be used as an independent prognostic factor for OS in patients with HCC.

### Construction of the Prognostic Nomogram

**FIGURE 5:**
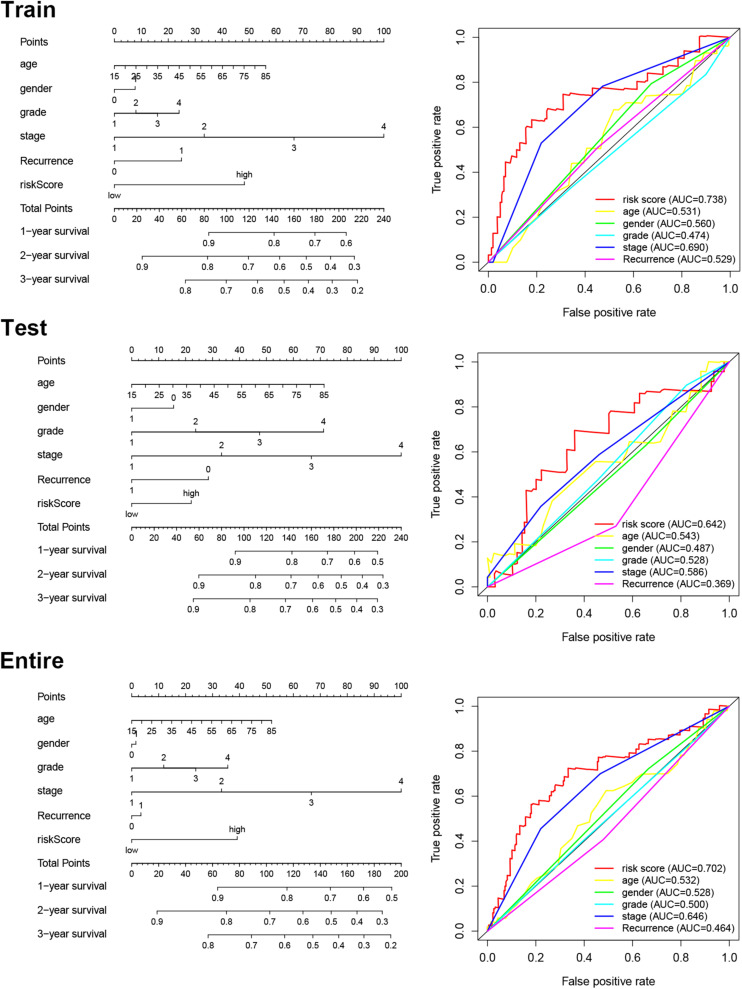
Nomogram for predicting OS and ROC curves of the risk scores and clinical features in each cohort.

We constructed a nomogram to predict OS in patients (311 HCC patients with known clinical characters) with HCC based on the risk score, age, gender, histological grade, pathological tumor stage, and recurrence (Figure 5). Subsequently, we used the ROC curve to assess the accuracy of the risk model (Figure 5). The computed AUC values of risk score and tumor stage were 0.738 and 0.690, respectively, in the training cohort, which are comparable to 0.642 and 0.586, respectively, in the testing cohort, and 0.702 and 0.646, respectively, in the entire cohort. The results suggest that the prognostic models constructed with the 5 immune-related eRNAs may be reliable predictors of OS in patients with HCC.

### The IReRS-Mediated Signaling Pathway in HCC

**FIGURE 6:**
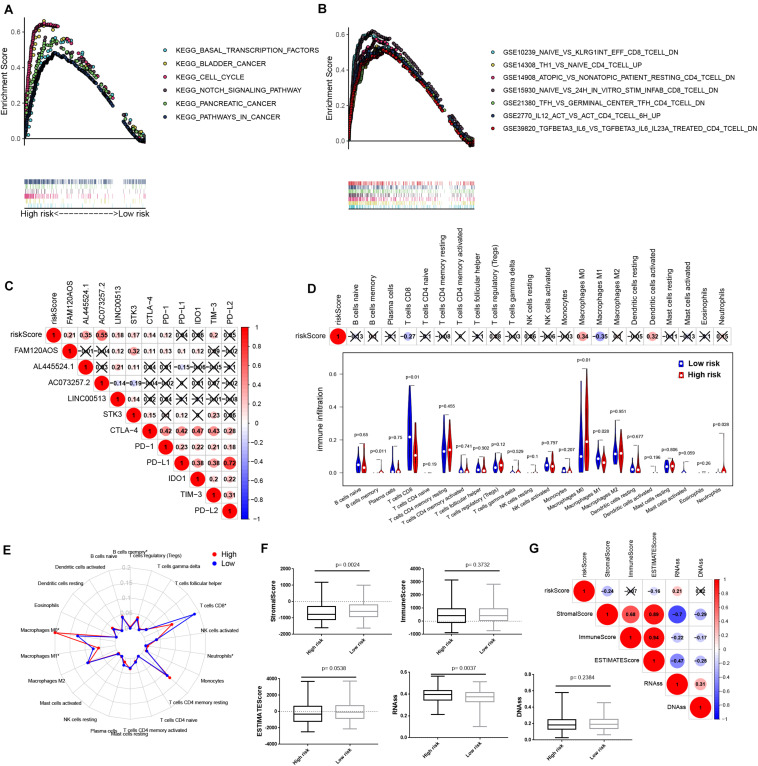
IReRS GSEA and relationship with immune checkpoint and immune cell infiltration. **(A)** Six cancer hallmarks were significantly enriched in the high-risk patients. **(B)** Seven immune signatures were significantly enriched in the high-risk patients. **(C)** Associations between immune-related eRNA signature and immune checkpoint genes were detected. Violin plot **(D)**, Radar map **(E)** were used to display the differences of the infiltrating immune cells in HCC patients between high- and low-risk groups. Boxplot **(F)** and correlation heat map **(G)** illustrated the correlations of risk score to immunoscore and tumor stem cell in HCC.

We next used GSEA to reveal the IReRS-mediated signaling pathway. As shown in Figure 6A, six cancer hallmarks were significantly enriched in high-risk HCC, including basal transcription factors, bladder cancer, cell cycle, notch signaling pathway, pancreatic cancer and pathways in cancer. Moreover, the GSEA results showed that high score of IReRS was also associated with numerous immune processes (Figure 6B), including GSE10239_NAIVE_VS_KLRG1NT_EFF_CD8_TCELL_DN, GS E14308_TH1_VS_NAIVE_CD4_TCELL_UP, GSE14908_ATO PIC_VS_NONATOPIC_PATIENT_RESTING_CD4_TCELL_DN, GSE15930_NAIVE_VS_24H_IN_VITRO _STIM_INFAB_CD8_ TCELL_DN, GSE21238_TFH_ VS_GERMINAL_CENTER_ TFH_CD4_TCELL_DN, GSE2770_IL12_ACT_VS_ACT_CD4_ TCELL_6H_UP, and GSE39820_TGFBETA3_IL6_VS_TGFBET A3_IL6_IL23A_TREATED_CD4_TCELL_DN These results indicated that IReRS may be involved in the progression of HCC by regulating the tumor immune microenvironment.

### The Relationship Between IReRS and Immune Cell Infiltration

The application of ICB for immunotherapy has become a promising aid to the treatment of various cancers, including HCC (Goodman et al., 2017; Kim et al., 2017; Nishino et al., 2017). Therefore, we investigated the possible role of our IReRS in the ICB therapy of HCC (Figure 6C) by evaluating the relationships of the six well known targets of immune checkpoint inhibitors (including TIM-3, IDO1, CTLA-4, PD-1, PD-L1, and PD-L2) to the IReRS and immune-related eRNAs. We found that the IReRS was minimally correlated to the expression of the six genes.

Next, we assessed the relationship between the IReRS and tumor immune microenvironment in HCC. Immune cell infiltration was obtained using CIBERSORT and the correlation between the IReRS risk score and immune cell infiltration was analyzed. We found that infiltration of CD8 T cells was negatively correlated with risk score, while the presence of macrophages M0 was positively correlated with risk score (Figures 6D,E). We next analyzed the correlation of the risk score to tumor microenvironment and tumor stem cell score in HCC, and found negative correlation with stromal score but positive correlation with RNAss (Figures 6F,G).

### Association of AL445524.1 With Poor Prognosis

**FIGURE 7:**
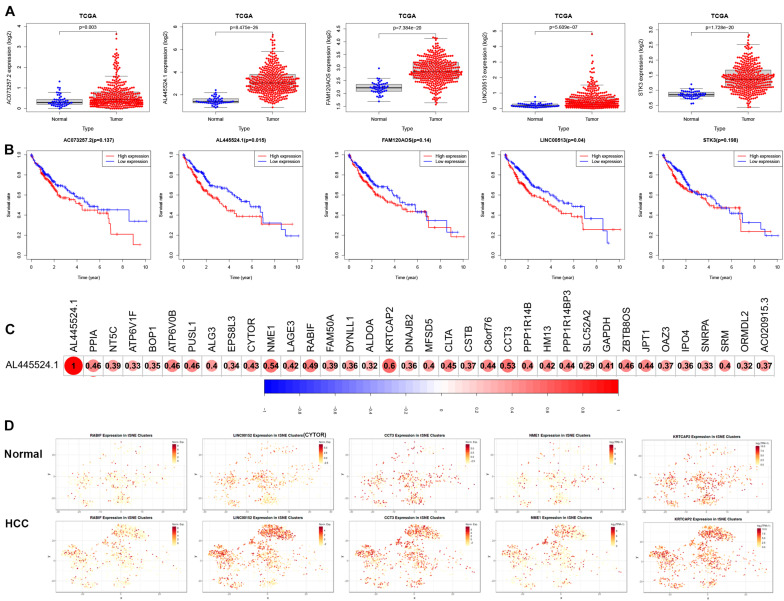
The expression of 5 modeled risk eRNAs and associated genes in HCC prognosis. **(A)** Risk eRNAs are differentially expressed between TCGA normal and HCC tissues. **(B)** Survival curves analyzed the role between high and low expression of risk eRNA. **(C)** *AL445524.1* is positively associated with 35 genes. **(D)** The tSNE analysis of *AL445524.1* associated five genes.

We next analyzed the expression and prognosis of the five modeled risk eRNAs. As shown in Figure 7A, all 5 risk eRNAs were significantly upregulated in HCC tissues compared to normal tissues. Moreover, both *AL445524.1* (*p* = 0.015) and *LINC00513* (*P* = 0.04) were associated with poor OS (Figure 7B). Further analyses of eRNA-related genes using Pearson correlation analysis with a significant level of R > 0.3 and *p* < 0.05 identified 35 genes to be positively associated with *AL445524.1* (Figure 7C), in which RABIF, CYTOR, CCT3, NME1, and KRTCAP2 were highly correlated with a significant level of R > 0.5 and *p* < 0.05. Then, we analyzed expression of the 5 eRNA-related genes in immune cells of HCC using tSNE cluster web tool (see text footnote 2). As shown in Figure 7D, the five genes were expressed more abundantly in the C8_CD4-CTLA4 bundle of HCC tissues than in normal tissues.

## Discussion and Conclusion

HCC is an immunologically-related malignancy, often arising from the context of persistent chronic inflammatory liver diseases, such as non-alcoholic fatty liver disease, chronic viral hepatitis B, and chronic viral hepatitis C (Champiat et al., 2016). The shift in the immune response from anti-tumor status to tumor tolerance contributes to the development and progression of HCC (Zamarron and Chen, 2011). Immune cells and immune-related genes have been reported as novel prognosis biomarkers and therapeutic targets for HCC (Jayant et al., 2020; Zhang et al., 2020). In this study, we aimed to construct a novel immune-related eRNA prognostic signature of HCC, and to characterize the eRNA signature as a useful prognostic tool and to identify therapeutic targets for HCC.

eRNAs have been found to be an important regulators of the immune response, and are involved in a mutiple tumorigenic signaling pathways, such as p53, PPARr, and immune checkpoints (Melo et al., 2013; Jayant et al., 2020), which are closely related to malignancy formation and progression. In this study, we constructed a five-gene IReRS as a new prognostic model and validated its predictive utility. On three datasets (the entire TCGA cohort and two subgroups), the predictive utility of the prognostic model was verified by Kaplan-Meier survival analyses and ROC curves. Both the clinical parameters and IReRS risk scores, in Cox regression analyses or constructed in a prognostic nomogram, predict the prognosis of HCC. More importantly, the predictive value of risk scores is superior to that of clinical parameters.

Next, the GSEA analysis showed that the IReRS was associated with multiple cancer related pathways and immune related pathways. Moreover, risk score was negatively correlated with CD8^+^ T cell and positively correlated with M0 macrophage infiltration in HCC. It was reported that an increase in CD8^+^ tumor infiltrating lymphocyte (TIL) was associated with improved overall survival in patients with HCC (Garnelo et al., 2017). Depletion of CD8^+^ TIL was found to be associated with the development of HCC (Wang et al., 2019).CD8 + T cells are important effector of TIL subsets in HCC, and play an important role in tumor immune surveillance and tumor eradication (Flecken et al., 2014). In contrast, immunosuppressive tumors associated macrophages (TAMs) are relevant to poor prognosis in HCC (Zhang Q. et al., 2019). Recently, accumulation of a large number of non-polarized M0 macrophages has been found in glioblastoma (Gabrusiewicz et al., 2016), and has been linked to tumor progression (Huang et al., 2020). In conclusion, risk score based on IReRS is an important predictor for the dysfunction of tumor immune microenvironment.

Finally, we analyzed the role and potential mechanism of 5 risk eRNAs. There have been limited PubMed reports on these five eRNAs in cancers, in which the *FAM120AOS* is highly expressed in colorectal cancer (Akbari et al., 2020) and glioma (Nikas, 2016); the *AC073257.2* targeting *GLI2*, affects the growth and proliferation of cellular keloids (Huang et al., 2018); and the *LINC00513* is identified in systemic lupus erythematosus (SLE) and associated with the type I interferon pathway by promoting phosphorylation of *STAT1* and *STAT2* proteins (Xue et al., 2018). In our study, all risk eRNAs were significantly upregulated in HCC tissues compared to adjacent normal tissues. Moreover, the risk gene *AL445524.1* was demonstrated to be associated significantly with poor prognosis of OS and immune-related genes including *RABIF, CYTOR, CCT3, NME1*, and *KRTCAP2.* The *RABIF* has been identified to be a biomarker candidate of breast cancer development (Switnicki et al., 2016) and associated with HCC (Li et al., 2021). The *CYTOR* has been reported being upregulated in many cancers, including HCC, lung adenocarcinoma, and renal cell carcinoma (Liang et al., 2018). The *CCT3* acts upstream of *YAP* and *TFCP2* as a potential target and tumour biomarker in liver cancer (Liu et al., 2019). The *NME1* promotes dynamin 2 oligomerization and regulated tumor cell endocytosis, motility, and metastasis (Khan et al., 2019). *KRTCAP2*, *MUC1*, and *TRIM46* are found to be highly expressed as chimeras in ovarian cancer (Kannan et al., 2015). Our study further shows that these 5 genes are highly expressed in the CD4-CLTA4 cluster of HCC. In a single-arm phase II study of 21 patients with advanced liver cancer, Child-Pugh type A or B, and/or hepatitis C, the anti-CTLA4 antibody tremelimumab is notably able to control the liver diseases (Sangro et al., 2013). Therefore, the *AL445524.1* may be used as an indicator for therapeutic choice of anti-CLTA4.

In summary, the immune-related eRNAs and modeled eRNA signature are independent prognostic biomarkers and are predictors of the immune microenvironment in HCC. Thus this study provides insights into a potentially novel predictive biomarker for the prognosis and survival of patients with HCC, and is expected to provide some possible options of improved immunotherapies.

## Data Availability Statement

The original contributions presented in the study are included in the article/supplementary material, further inquiries can be directed to the corresponding author/s.

## Author Contributions

SC and YZ: methodology and writing—original draft. XH and RC: investigation. XH, RC, SC, and YZ: writing—review and editing. All authors contributed to the article and approved the submitted version.

## Conflict of Interest

The authors declare that the research was conducted in the absence of any commercial or financial relationships that could be construed as a potential conflict of interest.

## Footnotes

1 http://genome.ucsc.edu

2 http://hcc.cancer-pku.cn/

## Abbreviations

AUC: the area under receiver operating characteristic curve
CTLA-4: cytotoxic T-lymphocyte antigen 4
GSEA: Gene Set Enrichment Analysis
HCC: hepatocellular carcinoma
ICB: immune checkpoint blockade
IReRS: immune-related eRNA signature
IRGs: immune-related genes
MDSC: myeloid-derived suppressive cells
OS: overall survival
PD-1: programmed death 1
PD-L1: programmed death ligand 1
PD-L2: programmed death ligand 2
PTGES2: prostaglandin E synthase 2
ROC: receiver operating characteristic
TAM: tumor associated macrophages
TCGA: The Cancer Genome Atlas
TIM-3: T-cell immunoglobulin domain and mucin domain-containing molecule-3.
